# Radiological Response and Characteristics of Patients Diagnosed With Hepatocellular Carcinoma Undergoing Transarterial Chemoembolization: An Observational Study

**DOI:** 10.1155/ijh/5821839

**Published:** 2025-09-21

**Authors:** Valeska A. Brito, Dayse C. B. L. Aroucha, Andressa M. Arruda, Ludmila C. C. Furtado, Victor P. Fonseca, Paulo C. R. Oliveira-Filho, Emília L. P. C. Branco, Maria F. B. L. Brito, Leila M. M. B. Pereira

**Affiliations:** ^1^Faculty of Medical Sciences, Institute of Biological Sciences, University of Pernambuco, Recife, Pernambuco, Brazil; ^2^Pernambuco Liver Institute, Recife, Pernambuco, Brazil; ^3^Faculty of Medical Sciences, University of Pernambuco, Recife, Pernambuco, Brazil

**Keywords:** hepatocellular carcinoma, interventional radiology, therapeutic chemoembolization

## Abstract

**Introduction:** Hepatocellular carcinoma (HCC), commonly associated with cirrhosis and factors such as viral hepatitis and metabolic disorders, is often diagnosed at advanced stages, influencing survival. Transarterial chemoembolization (TACE) is a primary therapeutic approach aimed at prolonging survival or serving as a link to liver transplantation.

**Objective:** To identify factors associated with the response to TACE by modified Response Evaluation Criteria in Solid Tumors (mRECIST) in patients with HCC.

**Materials and Methods:** This is a retrospective cohort study conducted at a Liver Institute in Brazil, including patients with HCC at Stages A and B treated with TACE from January 2011 to December 2021. Data were collected from electronic or digitized physical medical records and included demographic, clinical–laboratory, and tumor-related variables. Radiological response was assessed using mRECIST criteria. Statistical analysis encompassed various tests, with a significance level of 5%.

**Results:** Seventy-six patients were evaluated, the majority being male (67.1%), with a median age of 62 years (57.0–70.0). Patients who responded to TACE showed a significant reduction in lesion size (*p* < 0.001) compared to the nonresponding group, resulting in lesion enlargement (*p* = 0.047). Only 38.2% of patients showed an objective response after the first TACE, with a trend towards a higher response in patients with stable disease (*p* < 0.001). Hepatitis C virus (HCV) etiology was associated with a higher chance of treatment response (*p* = 0.032). Initial disease staging was characterized by single tumors, while intermediate staging presented larger tumors after TACE.

**Conclusion:** The association between HCV-induced cirrhosis and a better response to TACE underscores the importance of assessing liver function status in determining therapeutic response. No association was identified between pre-TACE alpha-fetoprotein levels and a higher likelihood of radiological response.

## 1. Introduction

Hepatocellular carcinoma (HCC) is the most common type of liver cancer, accounting for approximately 90% of primary liver cancers, and is classified as the sixth most common cancer worldwide and the third in terms of mortality [1, 2]. The risk of developing HCC is directly associated with the onset of liver cirrhosis, resulting in progressive impairment of liver function [3].

The majority of HCC cases manifest in patients with underlying liver pathologies, often associated with chronic viral hepatitis caused by viruses such as hepatitis B or C, chronic alcohol use, acquired or hereditary metabolic disorders such as nonalcoholic fatty liver disease (NAFLD), and genetic conditions such as hemochromatosis or alpha-1-antitrypsin deficiency [4, 5]. Among the aforementioned risk factors, there is a notable increase in the prevalence of NAFLD and nonalcoholic steatohepatitis (NASH), which may eventually surpass viral causes, resulting in the global development of HCC [6].

According to the clinical progression, HCC undergoes a prolonged subclinical growth period, during which therapeutic interventions can be applied with the potential for cure. However, most patients are diagnosed at intermediate or advanced stages of the disease, where curative therapeutic options are no longer obtainable [7, 8]. Therefore, expected survival varies according to the stage of the disease. In early stages, it exceeds 5 years in 40%–70% of cases, whereas in intermediate stages, it typically ranges between 20 and 30 months. Survival in advanced stages tends to be more limited, typically falling between 10 and 16 months [8, 9].

HCC diagnosis relies on noninvasive criteria, featuring a unique staging system historically resistant to conventional chemotherapy [8, 9]. Optimal treatment is determined by considering a complex interplay of factors, including tumor characteristics such as size, number, and location, as well as patient-related factors like liver function and overall health status [10]. Consequently, patients with early-stage HCC tumors are deemed preferable candidates for resection, transplantation, and local ablation, whereas those in intermediate stages are primarily directed towards transarterial chemoembolization (TACE). Conversely, patients with advanced disease typically undergo systemic therapies initially [2].

TACE is widely utilized in patients with unresectable HCC, either as a bridge to transplantation or as a recommended treatment to prolong survival in cases where resection or transplantation is not feasible [11]. TACE can be performed with chemotherapy emulsified in Lipiodol, followed by an embolic agent, such as gelatin sponge, polyvinyl alcohol particles (conventional TACE), or using pharmacological microspheres (DEB-TACE) [10, 12].

In addition, the modified Response Evaluation Criteria in Solid Tumors (mRECIST) for HCC aim to evaluate differences between necrosis and viable tumor on imaging studies, such as computed tomography (CT) or magnetic resonance imaging (MRI), following locoregional treatment such as TACE. Overall, mRECIST identifies two to three times more responders than standard RECIST in patients receiving locoregional treatments, as well as those undergoing systemic therapies [2]. Therefore, this study is aimed at identifying factors associated with response to TACE according to mRECIST criteria in patients with HCC.

## 2. Materials and Methods

### 2.1. Design, Setting, and Subjects

This is a retrospective single-center cohort study conducted at the Liver Institute in Brazil (*Instituto do Fígado de Pernambuco*). Inclusion criteria were patients, from both sexes, aged 18 years or older, diagnosed with HCC at Stages A and B according to the Barcelona Clinic Liver Cancer (BCLC) criteria. Diagnosis was confirmed through radiological criteria (contrast-enhanced CT or MRI) or liver biopsy. These patients underwent TACE between January 1, 2011, and December 31, 2021, and were seen at the hepatic nodules' outpatient clinic.

Data were obtained through a review of electronic or digitized physical medical records. Exclusion criteria were patients who received TACE before 2011, those who underwent any locoregional or surgical treatment prior to TACE, individuals with extrahepatic disease or intravascular invasion, those with BCLC Stage C or D, and those with incomplete or erroneous data.

### 2.2. Clinical Variables

Data from medical records were collected, including demographic factors such as sex and age at HCC diagnosis, pre-TACE clinical–laboratory parameters such as etiology of underlying liver disease, Child–Pugh scores, MELD-Na (Model for End-Stage Liver Disease plus serum sodium parameter), and ALBI (albumin–bilirubin) score, as well as alpha-fetoprotein (AFP) levels in nanograms per milliliter and BCLC staging (A or B). Tumor analysis included data on the number of tumors pre-TACE and the size of the target lesion before and after TACE in centimeters. All patients underwent contrast-enhanced imaging (CT or MRI) before and after TACE. Radiological response was assessed by collecting data from mRECIST reports in the medical records of the target lesion. Imaging analyses were performed by different radiologists due to logistical constraints in the local healthcare system, which made it impossible for the same radiologists to evaluate all the images. To minimize biases, the images were reviewed and discussed during multidisciplinary team meetings, which included all interventional radiologists. The primary outcome considered was the radiological response of the tumors.

### 2.3. Statistical Analyses

Data were analyzed using the Statistical Package for the Social Sciences (SPSS), Version 20.0 (SPSS Inc., Chicago, IL, United States). Categorical variables were presented as absolute (*n*) and relative frequencies (percentage) and compared using Pearson's chi-square test or Fisher's exact test. Normality of continuous variables was assessed using the Shapiro–Wilk test. Normally distributed data were expressed as mean and standard deviation, while non-normally distributed variables were presented as medians and interquartile ranges. Comparison analysis between two independent groups or three or more groups was performed using the Mann-Whitney test or Student's *t*-test and the Kruskal–Wallis test or ANOVA, respectively. Longitudinal comparison was assessed using the Wilcoxon test or paired Student's *t*-test. Binary logistic regression analysis was conducted to evaluate predictors. Statistical significance was established at 5%.

## 3. Results

**Figure 1:**
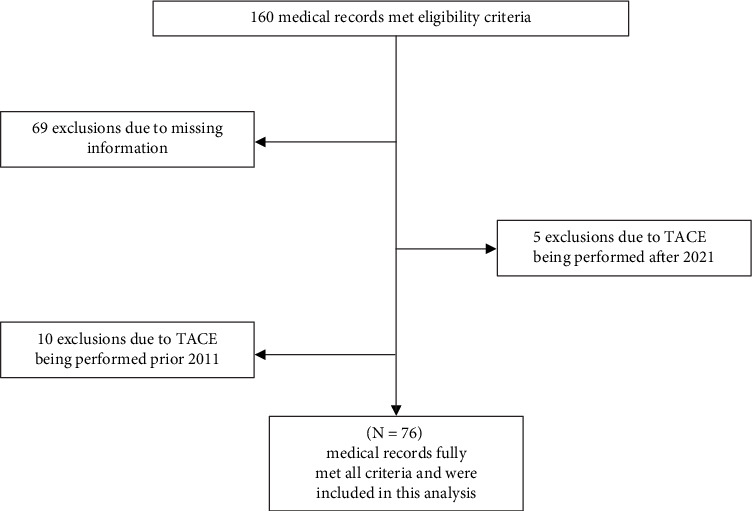
Study flowchart of medical record selection.

A total of 160 medical records of patients who met the inclusion criteria were screened. However, 69 patients were excluded due to missing information, five underwent TACE after 2021, and 10 patients underwent the procedure before 2011. Therefore, the sample comprised 76 patients followed during the initial and intermediate stages of the disease (Figure 1).

Of these, the highest frequency consisted of males (*n* = 51; 67.1%), with a median age of 62 years (57.0–70.0). Both males and females showed a higher frequency of involvement due to infectious causes, with 62.7% (95% CI 49.0–74.0) and 80.0% (95% CI 60.9–91.1), respectively. In terms of the lesion size pre- and post-TACE, females had a median of 5.1 (3.4–7.1) and 5.5 cm (2.6–7.5), respectively. Among males, the median size of the lesion pre-TACE was 4.5 cm (3.1–6.3), contrasting with a measurement of 3.0 cm (1.8–5.9) post-TACE. The highest frequency of only one tumor was observed in both groups, with 22 patients (43.1%; 95% CI 30.5–56.7) in males and 15 (60%; 95% CI 40.7–76.6) in females (Table 1).

**Figure 2:**
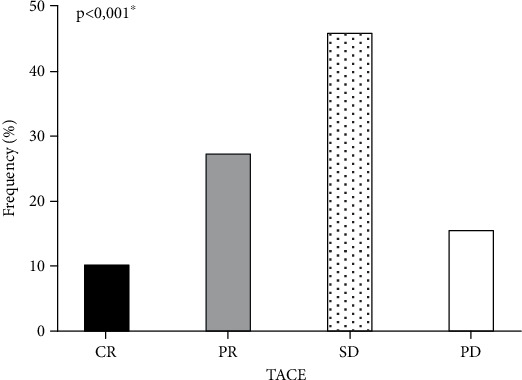
Comparison of response to TACE treatment using the mRECIST protocol. CR, complete response; PR, partial response; SD, stable disease; PD, progressive disease. Pearson's chi-square test was used to compare frequencies between groups. ⁣^∗^*p* < 0.05.

Univariate comparison of frequencies in response to TACE treatment using the mRECIST criteria protocol revealed that 10.5% (*n* = 8; 95% CI 5.4–19.4) of patients showed complete response (CR), 27.6% (*n* = 21; 95% CI 18.8–38.6) partial response (PR), 46.1% (*n* = 35; 95% CI 35.3–57.2) stable disease (SD), and 15.8% (*n* = 12; 95% CI 9.3–25.3) progressive disease (PD), emphasizing that only 38.2% (*n* = 29; 95% CI 28.1–49.4) showed objective response after the first TACE procedure. On the other hand, patients with SD had a better treatment response (*p* < 0.001), as demonstrated in Figure 2.

Upon analyzing the etiologies, a trend towards disparity between the groups that responded or did not respond to TACE treatment was observed (*p* = 0.053). Furthermore, there was an evident correlation between treatment response and lesion size variation following TACE. Patients who responded to treatment exhibited a reduction in lesion size, whereas those who did not respond showed an increase (*p* < 0.001) (Table 2).

**Figure 3:**
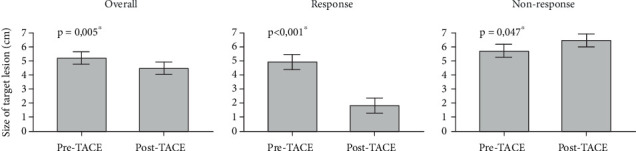
Size of target lesion (in centimeters) before and after TACE treatment. The Wilcoxon test was used to compare the longitudinal evolution of pre- and post-TACE lesion sizes for nonparametric variables. ⁣^∗^*p* < 0.05.

Further analysis, as depicted in Figure 3, unveiled the progression of lesion size in response to TACE, demonstrating that patients who responded to treatment experienced a notable reduction in lesion size (*p* < 0.001) compared to the nonresponder group, in which there was a documented increase in lesion size (*p* = 0.047).

When assessing the pre-TACE staging, a notable trend emerged: there was a higher identification but a lower number of tumors in the early stage (Stage A according to BCLC), with the majority of patients having only one tumor in this phase (*n* = 37; 90.2%). As the disease progressed (Stage B according to BCLC), the number of identified tumors increased, with a significant portion having two or more tumors (*n* = 21; 60%) (*p* < 0.001). Additionally, concerning lesion size, there was a significant difference noted post-TACE between the intermediate (Stage B) and initial stages (*p* < 0.001) (Table 3).

Upon analyzing the variables included in the binary logistic regression model concerning the response to TACE treatment, it was evident that hepatitis C virus (HCV) infection was significantly associated with an increased odds of treatment response (*p* = 0.032) (Table 4).

## 4. Discussion

TACE is a widely adopted therapeutic strategy for managing HCC, offering a relevant option for patients in advanced disease stages. Our analysis of the results has provided significant insights into the efficacy and determinants of therapeutic response within this population.

The prevalence of males and diagnoses above 50 years old was similar to other studies conducted in Brazil [13–16]. A systematic review by Nevola et al. [17] delved into factors linked to gender distribution in HCC, emphasizing a higher prevalence of risk factors among men, such as chronic viral hepatitis, liver cirrhosis, and excessive alcohol consumption. These disparities are attributed to behaviors, lifestyles, and biological factors, including hormonal variations that could impact the progression of liver disease.

According to etiology, the annual incidence of HCC in cases of HCV-induced cirrhosis ranges from 0.5% to 10% [18]. In this study, 51.3% of patients had cirrhosis attributed to HCV, aligning with data from studies across various regions, including Brazil, Latin America, the United States, Western Europe, and the Asia-Pacific [5, 13, 14, 19–21]. Conversely, in regions like Africa and East Asia, hepatitis B virus (HBV) is more prevalent, accounting for 60% of cases compared to the 20% observed in the Western world [5, 19]. This difference may be a consequence of socioeconomic, cultural, and public health differences, including limited access to healthcare, prevention programs, and vaccination, as well as cultural practices related to hygiene and the sharing of medical equipment [22].

In this study, we found an association between cirrhosis caused by HCV and a favorable response to TACE treatment. This finding may be attributed to the detection of HCC in its early stages, making it more manageable with TACE. Additionally, the slower progression of HCV-induced cirrhosis leads to better residual liver function, which increases treatment tolerance and decreases the risk of complications. Furthermore, patients may have received specific antiviral therapies targeting HCV, resulting in reduced viral load and further improvement in liver function, thereby enhancing the effectiveness of TACE [23].

Studies underscore the Child–Pugh score as a significant predictor of survival in patients undergoing TACE [24–27], despite its limitations in assessing ascites and encephalopathy [28]. Most patients in TACE studies present with Class A cirrhosis according to the Child–Pugh classification, indicating well-preserved liver function. Patients classified as Child–Pugh A are considered the ideal candidates for TACE, as emphasized by Bruix et al. [24]. In our study, 81.6% of patients were also classified as Child–Pugh A, suggesting that TACE during compensated cirrhosis should be the optimal timing to benefit from the procedure, compared to patients with more compromised liver function.

The median MELD-Na score observed in this study was 11, which is higher than that found in a study conducted by Kim et al. [29], which demonstrated a significant correlation between MELD and MELD-Na scores with survival in patients with HCC undergoing TACE. However, these scores were not considered superior to the Child–Pugh score.

This study also examined the ALBI score, introduced by Johnson et al. [30], as a specific scoring system for assessing liver function in HCC patients [28]. ALBI Grades 1 and 3 represent the best and worst survival, respectively [31]. Prior research, such as Mohammed et al. [32], showed that pre-TACE ALBI provided an objective prognosis for acute-on-chronic liver failure (ACLF) after TACE and could aid in risk stratification. Moreover, a Japanese–German study [33] associated the ALBI score with lower progression-free survival, highlighting the importance of liver function in overall and progression-free survival [34]. In this study, approximately 56% of patients were classified as ALBI Grade 2, indicating moderate impairment of liver function. This higher prevalence of patients with Child–Pugh A classification and ALBI Grade 2 may be explained by their relatively preserved liver function, rendering them more responsive to TACE and potentially leading to a better treatment outcome.

AFP remains the primary biomarker for screening, despite controversies regarding its clinical effectiveness. Elevations in AFP levels may indicate adverse outcomes and advanced tumor stage at the time of diagnosis [35]. In our study, the median AFP before TACE treatment was 24.4 ng/mL, while Appel-da-Silva et al. [14] reported a median below 20 ng/mL, and in Carrilho et al. [13], the majority of patients had AFP < 100 ng/mL. This variation may be influenced by population characteristics, disease stage, detection methods, and previous treatment.

An important observation in our study was that the majority of patients had fewer than four tumors before TACE therapy, with 48.7% presenting with only one tumor. However, most had two or more nodules, consistent with findings from a randomized clinical trial conducted by Llovet et al. [36]. Additionally, the median lesion size before the first TACE session was 4.8 cm, which was higher than the median reported in another study conducted in Korea (3.1 cm) [37]. Larger (> 5 cm) and multiple tumors (> 3) were associated with a lack of CR after the first TACE (*p* < 0.001), underscoring the significance of assessing tumor size and number as predictors of TACE efficacy in HCC.

In our study, the majority of patients (46.1%) exhibited SD after the first TACE therapy, while only 10.5% achieved a CR. In contrast, Peng et al. [38] reported CR, PR, SD, and PD rates of 22.3%, 32.9%, 18.8%, and 26.0%, respectively. Patients who attained CR after the initial TACE demonstrated a significantly longer median overall survival compared to those without CR (35.8 and 24.0 months, respectively; *p* < 0.001). Factors such as Child–Pugh Class B, bilobar tumor extent, elevated AFP (≥ 20 ng/mL), and higher platelet counts (> 150,000/*μ*L) were identified as unfavorable predictors for CR following the initial TACE.

In the study by Georgiades et al. [39], 58 patients, corresponding to 50% of the sample, did not respond to the first TACE. However, 27 of these patients responded to the second TACE. This suggests that the lack of response to the first TACE does not preclude the possibility of responding to the second one. Although our study did not address evaluations of the second TACE, the results indicate that patients with HCC may benefit from multiple TACE sessions to improve survival.

These findings suggest that TACE was effective in controlling the disease in a significant proportion of the sample, with disease stabilization potentially bringing important clinical benefits such as improved quality of life and increased survival. The high rate of SD after the first TACE underscores the importance of this treatment as a therapeutic option for patients with HCC, especially those ineligible for surgery or liver transplantation.

In the current study, it was also observed that the size of the target lesion varied depending on the treatment response. Patients who achieved PR or CR, indicating objective response, demonstrated a reduction in size after the first TACE. These results align with existing literature, which similarly suggests that nonresponsive patients experience lesion enlargement [39, 40].

When comparing demographic, clinical–laboratory, and tumor-related variables with BCLC staging pre-TACE, we observed that only the number of tumors pre-TACE showed statistical significance. This is attributable to the inherent structure of the BCLC classification, which determines patient stage based on parameters such as tumor size and number, associating stage with the proposed treatment.

Our study had several limitations, including its retrospective design and the small sample size, representing a significant constraint. Moreover, the single-center sample restricts the generalizability of our findings to broader populations.

## 5. Conclusion

The findings underscore the effectiveness of TACE in managing HCC, as most patients achieved disease stability following initial treatment. The link between HCV-induced cirrhosis and improved TACE response highlights the crucial role of assessing hepatic function in predicting therapeutic outcomes. Furthermore, prognostic factors such as Child–Pugh and ALBI scores are discussed in terms of their impact on treatment response. These insights offer valuable guidance for clinical decision-making and treatment strategies for HCC, emphasizing the importance of personalized approaches based on patient and tumor characteristics to enhance treatment efficacy.

## Figures and Tables

**Table 1:** Characterization of patients with hepatocellular carcinoma according to demographic, clinical–laboratory, and tumor variables (*N* = 76).

**Variables**	**Median (IQ) or** **N** **(%)**	**95% CI**
Sex^a^		
Males	51 (67.1)	55.9–76.6
Females	25 (32.9)	23.4–44.0
Age (years)^b^	64.0 (58.2–71.0)	—
Cirrhosis etiology^a^		
HBV	8 (10.5)	5.4–19.4
HCV	39 (51.3)	40.3–62.2
Alcoholic	11 (14.5)	8.3–24.1
Others	18 (23.7)	15.5–34.3
Pre-TACE Child^a^		
A	62 (81.6)	71.4–88.7
B	14 (18.4)	11.3–28.6
Pre-TACE MELD-Na^b^	11.0 (9.0–14.0)	—
Pre-TACE BCLC^a^		
A	41 (53.9)	42.8–64.7
B	35 (46.1)	35.3–57.2
Pre-TACE AFP^b^ (ng/mL)	24.4 (4.8–306.2)	—
Pre-TACE ALBI^a^		
1	30 (39.5)	29.2–50.7
2	43 (56.6)	45.4–67.1
3	3 (3.9)	1.3–10.9
Pre-TACE number of tumors^a^		
1	37 (48.7)	37.8–59.7
2	23 (30.3)	21.1–41.3
3	8 (10.5)	5.4–19.4
4	4 (5.3)	2.1–12.7
> 4	4 (5.3)	2.1–12.7
Pre-TACE target lesion size (cm)^b^	4.8 (3.4–6.6)	—
Post-TACE target lesion size (cm)^b^	3.6 (2.0–6.4)	—

*Note:* Data were expressed as (a) absolute (*N*) and relative frequency (%) and (b) median (IQ).

Abbreviations: 95% CI, 95% confidence interval; AFP, alpha-fetoprotein; ALBI, albumin–bilirubin score; BCLC, Barcelona Clinic Liver Cancer; Child, Child–Pugh score; cm, centimeters; HBV, hepatitis B virus; HCV, hepatitis C virus; IQ, interquartile range; MELD-Na, model for end-stage liver disease plus sodium; TACE, transarterial chemoembolization.

**Table 2:** Comparison between demographic, clinical–laboratory, and tumor variables with response to TACE in patients with hepatocellular carcinoma (*N* = 76).

**Variables**	**TACE response**						
	**Total**		**Objective response (CR e PR)**		**No objective response (SD e PD)**		**p** **value**
	**N**	**%**	**N**	**%**	**N**	**%**	
Sex^a^							
Males	51	67.1	21	72.4	30	63.8	0.439
Females	25	32.9	8	27.6	17	36.2	
Age (years)^b^	64.0 (58.2–71.0)		64.0 (59.0–70.5)		64.0 (58.0–71.0)		0.638
Cirrhosis etiology^a^							
HBV	8	10.5	1	3.4	7	14.9	0.053
HCV	39	51.3	17	58.6	22	46.8	
Alcoholic	11	14.5	7	24.1	4	8.5	
Others	18	23.7	4	13.8	14	29.8	
Pre-TACE Child^a^							
A	62	81.6	24	82.8	38	80.9	0.835
B	14	18.4	5	17.2	9	19.1	
Pre-TACE MELD-Na^b^	11.0 (9.0–14.0)		11.0 (8.5–12.5)		11.0 (9.0–15.0)		0.579
Pre-TACE BCLC^a^							
A	41	53.9	15	51.7	26	55.3	0.760
B	35	46.1	14	48.3	21	44.7	
Pre-TACE AFP^b^ (ng/mL)	24.4 (4.8–306.2)		9.3 (3.4–239.2)		25.4 (6.7–401.0)		0.155
Pre-TACE ALBI^a^							
1	30	39.5	13	44.8	17	36.2	0.754
2	43	56.6	15	51.7	28	59.6	
3	3	3.9	1	3.4	2	4.3	
Pre-TACE number of tumors^a^							
1	37	48.7	14	48.3	23	48.9	0.537
2	23	30.3	7	24.1	16	34.0	
3	8	10.5	3	10.3	5	10.6	
4	4	5.3	3	10.3	1	2.1	
> 4	4	5.3	2	6.9	2	4.3	
Pre-TACE target lesion size (cm)^b^	4.8 (3.4–6.6)		4.3 (3.5–7.2)		5.1 (3.2–7.2)		0.237
Post-TACE target lesion size (cm)^b^	3.6 (2.0–6.4)		1.8 (0–2.6)		5.9 (3.6–8.0)		< 0.001⁣^∗^

*Note:* Data were expressed as (a) absolute frequency (*N*) and relative (%) and (b) median (IQ). (a) Pearson's chi-square test. (b) Mann-Whitney test.

Abbreviations: 95% CI, 95% confidence interval; AFP, alpha-fetoprotein; ALBI, albuminbilirubin score; BCLC, Barcelona Clinic Liver Cancer; Child, Child–Pugh score; cm, centimeters; CR, complete response to TACE; HBV, hepatitis B virus; HCV, hepatitis C virus; IQ, interquartile range; MELD-Na, model for end-stage liver disease plus sodium; PD, progressive disease; PR, partial response to TACE; SD, stable disease; TACE, transarterial chemoembolization.

⁣^∗^*p* < 0.05.

**Table 3:** Comparison between demographic, clinical–laboratory, and tumor variables with BCLC staging pre-TACE in patients with hepatocellular carcinoma (*N* = 76).

**Variables**	**Pre-TACE BCLC**						
	**Overall**		**A**		**B**		**p** **value**
	**N**	**%**	**N**	**%**	**N**	**%**	
Sex^a^							
Males	51	67.1	26	63.4	25	71.4	0.459
Females	25	32.9	15	36.6	10	28.6	
Age (years)^b^	64.0 (58.2–71.0)		65.0 (59.0–71.0)		63.0 (58.0–71.0)		0.638
Cirrhosis etiology^a^							
HBV	8	10.5	4	9.8	4	11.4	0.776
HCV	39	51.3	23	56.1	16	45.7	
Alcoholic	11	14.5	6	14.6	5	14.3	
Others	18	23.7	8	19.5	10	28.6	
Pre-TACE Child^a^							
A	62	81.6	33	80.5	29	82.9	0.791
B	14	18.4	8	19.5	6	17.1	
Pre-TACE MELD-Na^b^	11.0 (9.0–14.0)		11.0 (8.5–15.0)		11.0 (9.0–14.0)		0.579
Pre-TACE AFP^b^ (ng/mL)	24.3 (4.8–306.8)		8.8 (3.6–237.5)		45.0 (9.3–680.0)		0.155
Radiological response^a^							
Response (CR e PR)	29	38.2	15	36.6	14	40.0	0.760
Nonresponse (SD e PD)	47	61.8	26	63.4	21	60.0	
Pre-TACE ALBI^a^							
1	30	39.5	17	41.5	13	37.1	0.739
2	43	56.6	23	56.1	20	57.1	
3	3	3.9	1	2.4	2	5.7	
Pre-TACE number of tumors^a^							
1	37	48.7	37	90.2	0	0	< 0.001⁣^∗^
2	23	30.3	2	4.9	21	60.0	
3	8	10.5	2	4.9	6	17.1	
4	4	5.3	0	0	4	11.4	
> 4	4	5.3	0	0	4	11.4	
Pre-TACE target lesion size (cm)^b^	4.8 (3.4–6.6)		5.8 (3.0–7.5)		4.5 (3.6–5.1)		0.237
Post-TACE target lesion size (cm)^b^	3.6 (2.0–6.4)		3.5 (1.9–7.2)		3.6 (2.1–5.6)		< 0.001⁣^∗^

*Note:* A: Barcelona Clinic Liver Cancer (BCLC) Stage A; B: Barcelona Clinic Liver Cancer (BCLC) Stage B. Data are presented as (a) absolute frequency (*N*) and relative (%) and (b) median (IQ). (a) Pearson's chi-square test. (b) MannWhitney test.

Abbreviations: 95% CI, 95% confidence interval; AFP, alpha-fetoprotein; ALBI, albuminbilirubin score; Child, ChildPugh score; cm, centimeters; CR, complete response to TACE; HBV, hepatitis B virus; HCV, hepatitis C virus; IQ, interquartile range; MELD-Na, model for end-stage liver disease plus sodium; PD, progressive disease; PR, partial response to TACE; SD, stable disease; TACE, transarterial chemoembolization.

⁣^∗^*p* < 0.05.

**Table 4:** Binary logistic regression analysis of demographic, clinical–laboratory, and tumor variables according to TACE response in patients with hepatocellular carcinoma (*N* = 76).

**Variables**	**β** ^∗^	**OR**	**95% CI**	**p** **value**
Age (years)	−0.011	0.99	0.94–1.03	0.633
Males	−0.397	0.67	0.24–1.84	0.440
HCV	1.812	6.12	1.17–32.1	0.032⁣^∗^
Pre-TACE Child A	−0.128	0.88	0.26–2.94	0.835
Pre-TACE MELD-Na	0.045	1.05	0.92–1.20	0.489
Pre-TACE BCLC A	0.145	1.16	0.46–2.92	0.760
Pre-TACE AFP (ng/mL)	0.000	1.00	1.00–1.00	0.446
Pre-TACE ALBI	−0.425	0.66	0.05–8.02	0.740
Pre-TACE number of tumors	0.496	1.64	0.21–13.01	0.638
Pre-TACE target lesion size (cm)	0.138	1.15	0.94–1.41	0.185
Post-TACE target lesion size (cm)	1.072	2.92	1.74–4.92	< 0.001⁣^∗^

Abbreviations: *β*^∗^, standardized coefficient; 95% CI, 95% confidence interval; AFP, alpha-fetoprotein; ALBI, albumin–bilirubin score; BCLC, Barcelona Clinic Liver Cancer; Child, Child–Pugh score; cm, centimeters; HCV, hepatitis C virus; MELD-Na, Model for End-Stage Liver Disease plus sodium; OR, odds ratio; TACE, transarterial chemoembolization.

⁣^∗^*p* < 0.05.

## Data Availability

Data used as the basis for reaching the conclusions of this study are included in the article.
